# Global Effects of Focal Brain Tumors on Functional Complexity and Network Robustness: A Prospective Cohort Study

**DOI:** 10.1093/neuros/nyy378

**Published:** 2018-08-22

**Authors:** Michael G Hart, Rafael Romero-Garcia, Stephen J Price, John Suckling

**Affiliations:** 1 Brain Mapping Unit, Department of Psychiatry, Sir William Hardy Building, University of Cambridge, Cambridge, United Kingdom; 2 Academic Division of Neurosurgery, Department of Clinical Neurosciences, University of Cambridge, Cambridge, United Kingdom; 3 Brain Mapping Unit, Department of Psychiatry, Herchel Smith Building for Brain and Mind Sciences, University of Cambridge, Cambridge, United Kingdom

**Keywords:** Connectome, Fractal, Functional MRI, Glioblastoma, Neurooncology

## Abstract

**BACKGROUND:**

Neurosurgical management of brain tumors has entered a paradigm of supramarginal resections that demands thorough understanding of peritumoral functional effects. Historically, the effects of tumors have been believed to be local, and long-range effects have not been considered.

**OBJECTIVE:**

To test the hypothesis that tumors affect the brain globally, producing long-range gradients in cortical function.

**METHODS:**

Resting-state functional magnetic resonance imaging (fMRI) data were acquired from 11 participants with glioblastoma and split into discovery and validation datasets in a single-center prospective cohort study. Fractal complexity was computed with a wavelet-based estimator of the Hurst exponent. Distance-related effects of the tumors were tested with a tumor mask-dilation technique and parcellation of the underlying Hurst maps.

**RESULTS:**

Fractal complexity demonstrates a penumbra of suppression in the peritumoral region. At a global level, as distance from the tumor increases, this initial suppression is balanced by a subsequent overactivity before finally normalizing. These effects were best fit by a quadratic model and were consistent across different network construction pipelines. The Hurst exponent was correlated with graph theory measures of centrality including network robustness, but graph theory measures did not demonstrate distance-dependent effects.

**CONCLUSION:**

This work provides evidence supporting the theory that focal brain tumors produce long-range gradients in function. Consequently, the effects of focal lesions need to be interpreted in terms of the global changes on functional complexity and network architecture rather than purely in terms of functional localization. Determining whether peritumoral changes represent potential plasticity may facilitate extended resection of tumors without functional cost.

**ABBREVIATIONS**
ANCOVA: analysis of covariance
ANOVA: analysis of variance
ANTs: advanced normalization tools
BOLD: blood oxygenation level-dependent
fMRI: functional magnetic resonance imaging
ME-ICA: multiecho independent component analysis

A new philosophy of supra-marginal resection has entered the vocabulary of neuro-oncology whereby surgical excision is extended beyond the confines of a lesion as it is visible on standard structural imaging. This paradigm is based on the identification of tumor infiltration beyond the contrast-enhancing margin,^1-4^ fluorescence-guided imaging that can identify potentially infiltrated brain at surgery,^5,6^ clinical studies correlating extended resections with improved survival,^7-10^ and potentially improved effectiveness of adjuvant therapies.^11^ However, extending surgery outside the lesion margins comes with the risk of intruding on eloquent brain and consequently the potential of neurological impairment. Therefore, understanding the function of the peritumoral brain is of paramount importance.

This requirement to accurately map brain function led to many notable contributions from neurosurgery in understanding functional neuroanatomy.^12,13^ However, the traditional approach to understanding the effects of focal tumors per se on brain function is somewhat limited. Firstly, the effects of brain lesions on the surrounding tissue are believed to be only local (ie, through structural disruption), and potential long-range effects are overlooked. Furthermore, function itself is viewed as being uniform and consequently represented as a binary outcome (ie, brain either is or is not eloquent); considering function as a continuous variable has not been investigated. Understanding putative long-range effects of brain tumors and nonbinary gradients in function may allow more accurate prediction of the effects of tumor removal and insight into mechanisms of plasticity-related recovery.

Fractal analysis of resting-state functional magnetic resonance imaging (fMRI) data offers the potential to understanding putative long-range gradients in brain function. Fractals are signals that display scale invariance; that is, they have similar features regardless of the scale at which they are viewed. Fractal signals are pervasive in nature (examples include coastlines, snowflakes, and ocean waves) and in the brain (neuronal membrane potentials, neural field potentials as well as electroencephalography, magnetoencephalography, and blood oxygenation level-dependent [BOLD] contrast endogenous signals).^14^ From a fractal perspective, healthy homeostasis emphasizes the complexity of the underlying biological processes rather than regular steady-state behavior.^15,16^ In the brain, this complexity is believed to enable adaptation to stimuli or other challenges, while simpler and less complex dynamics are indicative of less advantageous function or disease.^17-20^

Connectomics is a novel multidisciplinary paradigm that naturally views the brain as a complex network of individual components interacting through continuous communication and offers a window into the effects of focal tumors at the global level.^21^ Furthermore, graph theory analysis of brain networks unlocks a new terminology with which to describe brain functional neuroanatomy and for predictive modeling of, for example, the effects of tumor removal and how this is related to network robustness.^22^ Combining fractal and network analysis methods provides a complimentary approach to identifying putative wide-ranging effects of focal brain tumors on signal complexity and network robustness.

In this work, the concept that tumors only lead to local effects on brain function is challenged, and a new theory that considers the global effects of a tumor is proposed that can augment our current brain mapping techniques. BOLD sensitive, resting-state fMRI was used to map brain function with data-driven analysis of fractal complexity and functional connectivity at the whole brain level. It was hypothesized that tumors would produce long-range effects outside of their margin as depicted on structural magnetic resonance imaging (MRI) and that there would be gradients of BOLD signal and functional connectivity extending away from the tumor. Evidence is presented to support the theory that focal brain tumors produce long-range and nonlinear gradients in function.

## METHODS

### Design

The study was approved by the Local Regional Ethics Committee (protocol number10/H0308/23) and was a single-center prospective cohort design. All participants provided written informed consent. Inclusion criteria were the MRI appearance of a tumor consistent with a glioblastoma. All participants had a confirmed glioblastoma at local histological review according to World Health Organization criteria.^23^ Resective surgery was performed in all cases under general anesthesia using an operating microscope (OPMI Pentero 900, Carl Zeiss Meditec AG, Oberkochen, Germany) with fluorescence guidance (Gliolan®, medac Pharma, Stirling, United Kingdom) and neuronavigation (StealthStation® S7® System, Medtronic Inc, Dublin, Ireland).

A cohort of 5 participants was selected to form a discovery dataset for hypothesis generation. Subsequently, a further 6 participants were included, forming the validation dataset. The discovery dataset was designed to be homogeneous and included moderately sized tumors based on the right parietal lobe, whereas the validation dataset was designed to be more heterogeneous with a variety of tumor sizes and locations. Splitting the group into separate discovery and validation cohorts was performed to test if findings were replicable across a variety of tumor presentations. Demographic information is summarized in Table 1.

**TABLE 1.** Participant Demographics.

Age	Sex	Seizures	Pre-op exam	Post-op exam	Pathology	Surgery	Tumor location	Molecular markers	Survival (months)
64	F	Pre-op	Left pronator drift	Homonymous hemianopia	Glioblastoma	No residual disease	Right superior parietal lobule	Negative/85%	23
73	F	No	Intact	Homonymous hemianopia	Glioblastoma	No residual disease	Right inferior parietal lobule to occipital pole	Negative/MIB 32%	13
79	M	No	Hemianopia	No change	Glioblastoma	No residual disease	Right inferior occipital lobe	Negative/MIB 28%	17
76	M	Pre-op	Left hemiparesis	Paresis deterioration	Glioblastoma	Biopsy	Right superior paracentral lobule	Negative/MIB 18%	Unavailable
36	M	Post-op	Left hemiparesis	No change	Anaplastic astrocytoma	No residual disease	Right postcentral gyrus and supramarginal gyrus	Negative/MIB 30%	
62	M	No	Hemianopia	No change	Glioblastoma	Biopsy	Right thalamic extending to temporo-parietal-occipital region	Negative/MIB 12.5%	14*
52	M	No	Left hemiparesis	No change	Glioblastoma	Complete resection	Right supramarginal gyrus	Negative/MIB 25%	15*
57	M	No	Memory and language	No change	Glioblastoma	Complete resection	Left superior frontal gyrus	Negative/MIB 26.1%	16*
36	M	No	Hemianopia	No change	Glioblastoma	Complete resection	Right parieto-occipital	Negative/MIB 8.3%	18*
44	M	Pre-op	Nil	No change	Glioblastoma	Biopsy	Left caudate	IDH-1 & MGMT positive/ MIB 38%	12*
50	M	No	Hemianopia	No change	Glioblastoma	Complete resection	Right supramarginal gyrus/occipital lobe	Negative/MIB 45%	8

The first 5 participants formed the discovery dataset, and the subsequent 6 participants formed the validation dataset. Extent of resection is based on RANO criteria.^24^ Molecular markers include MGMT promoter methylation, IDH-1 mutation, and 1p19q status. MIB-1 = mindbomb antibody. *Censored data.

### Imaging

MRI data were acquired using a Siemens Trio 3T scanner and 16-channel receive-only head coil (Siemens AG, Erlangen, Germany). A multiecho echo-planar imaging sequence was performed for 10 min and 51 s at a repetition time, TR = 2.42 s resulting in 269, 3-dimensional volumes covering the cerebral cortices and cerebellum. Anatomical images were acquired using a T1-weighted magnetization prepared rapid gradient echo sequence.

**FIGURE 1.**
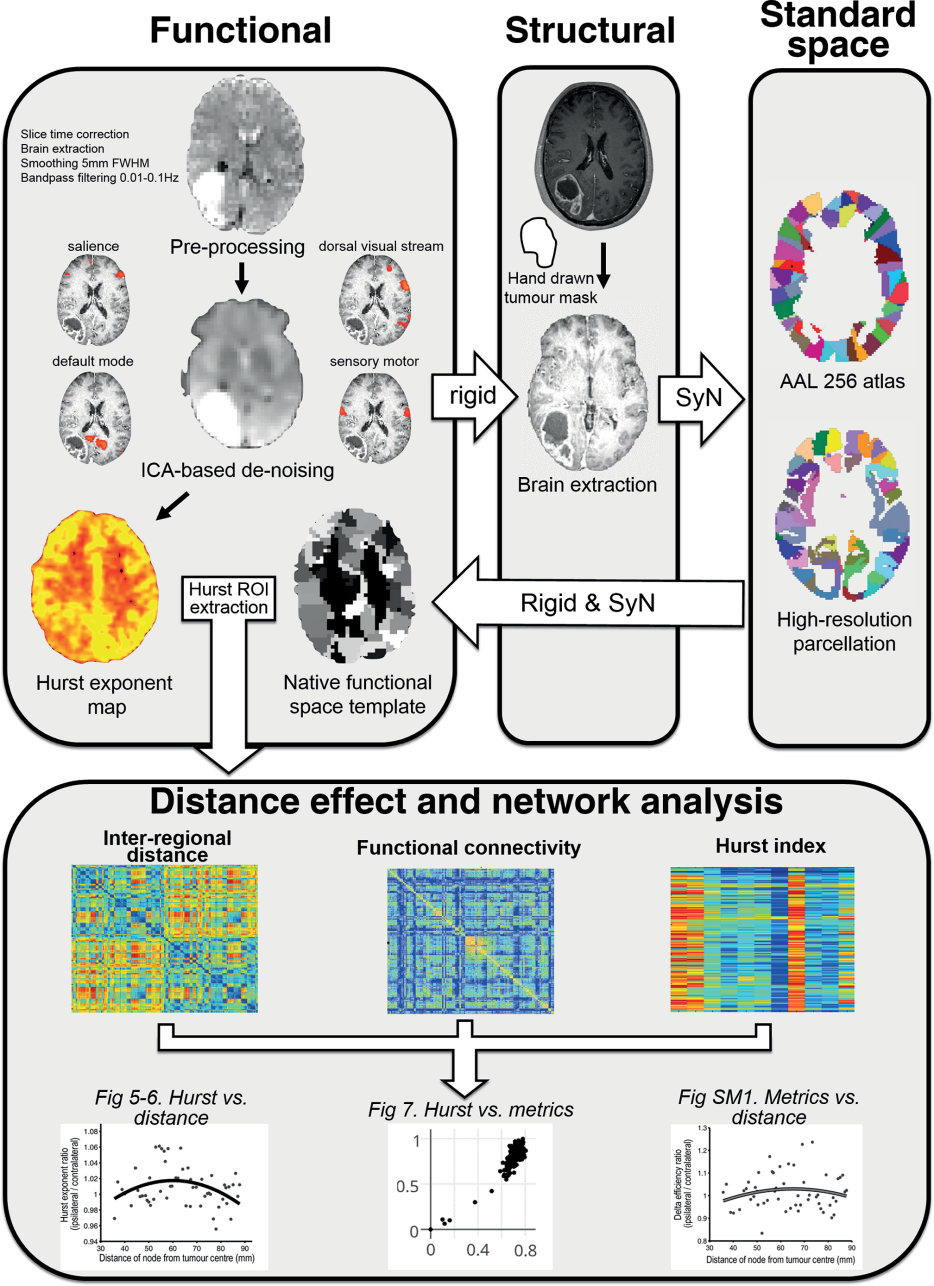
Structural and functional image analysis pipeline. Functional images underwent multiecho ICA-based denoising and selected prestatistical processing in functional space. Anatomical images underwent brain extraction, bias field correction, and tissue segmentation (including hand drawn masks of the tumor contrast-enhancing volume). Standard space parcellation templates were warped to the structural image using symmetrical diffeomorphic registration and a binary tumor mask to exclude the region from cost function weighting. An additional rigid 6 degree of freedom transform was performed from structural to functional space, and then these transforms were combined to produce a representation of the parcellation template in functional space, obviating the need for interpolation of the functional image. Fractal analysis was performed in structural space to allow optimal delineation of the tumor mask. SyN = symmetrical diffeomorphic normalisation. FWHM = fixed width at half maximum.

A summary of the analysis pipeline is presented in Figure 1. Advanced Normalization Tools (ANTs) cortical thickness pipelines were used for structural analysis (http://stnava.github.io).^25-27^ A hand drawn tumor mask to encompass the contrast-enhancing margin was created by the neurosurgeon and used as an additional prior for segmentation. Finally, images were nonlinearly mapped to the space of the Montreal Neurological Institute using symmetrical diffeomorphic registration with masking of the tumor from cost-function calculation.

Data preprocessing was performed using AFNI (http://afni.nimh.nih.gov/afni/).^28^ For denoising, multiecho independent component analysis (ME-ICA) was used.^29^ Linear registration matrices (6 degrees of freedom) between functional and structural acquisition space were calculated with ANTs.

### Fractal Analysis

**FIGURE 2.**
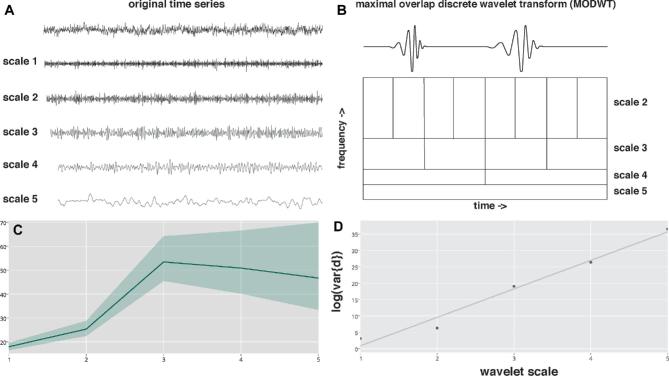
Fractal analysis and hurst exponent calculation. **A**, The time series of BOLD contrast for a selected brain region (in this case the right superior frontal gyrus) is displayed on top: underneath are the same time series at wavelet scales 1 to 5. **B**, The maximal overlap discrete wavelet transform (MODWT) scales the wavelet in frequency and amplitude to decompose the original time series at multiple scales (note the planned decomposition is also a natural fractal). Here we illustrate Daubechies 8 wavelet (note it integrates to 0 and has an irregular shape) at two scales, above. **C**, Wavelet variance of the decomposed time series scales plotted against scale. As wavelet scale increases (and therefore frequency decreases) the variance increases. **D**, Self-similarity of variance is exponentially related to the scale and therefore forms a straight line on a plot of wavelet scale versus the log of the variances of the wavelet (detail) coefficients at the corresponding scale, log(var{d}). The exponent of the straight line is related to the Hurst exponent ($H\ = \ \frac{{\gamma + 1}}{2})$ and thereafter the fractal dimension (D = 2 − H).

Fractal analysis was performed with a wavelet-based estimator of the Hurst exponent (Figure 2) applied to processed resting state fMRI data registered to the corresponding structural image.^30^ In brief, time series at each voxel underwent wavelet decomposition. A linear model was regressed on to the logarithm of the variance of wavelet coefficients as a function of scale. The estimated slope of this model is proportional to the Hurst exponent, H, and in turn the fractal dimension. Hurst exponents were obtained for every intracerebral voxel, and then thresholded at H <0.5 (corresponding to white noise) and values were extracted for each parcel of the parcellation template (see below).

### Peritumoral Analysis

**FIGURE 3.**
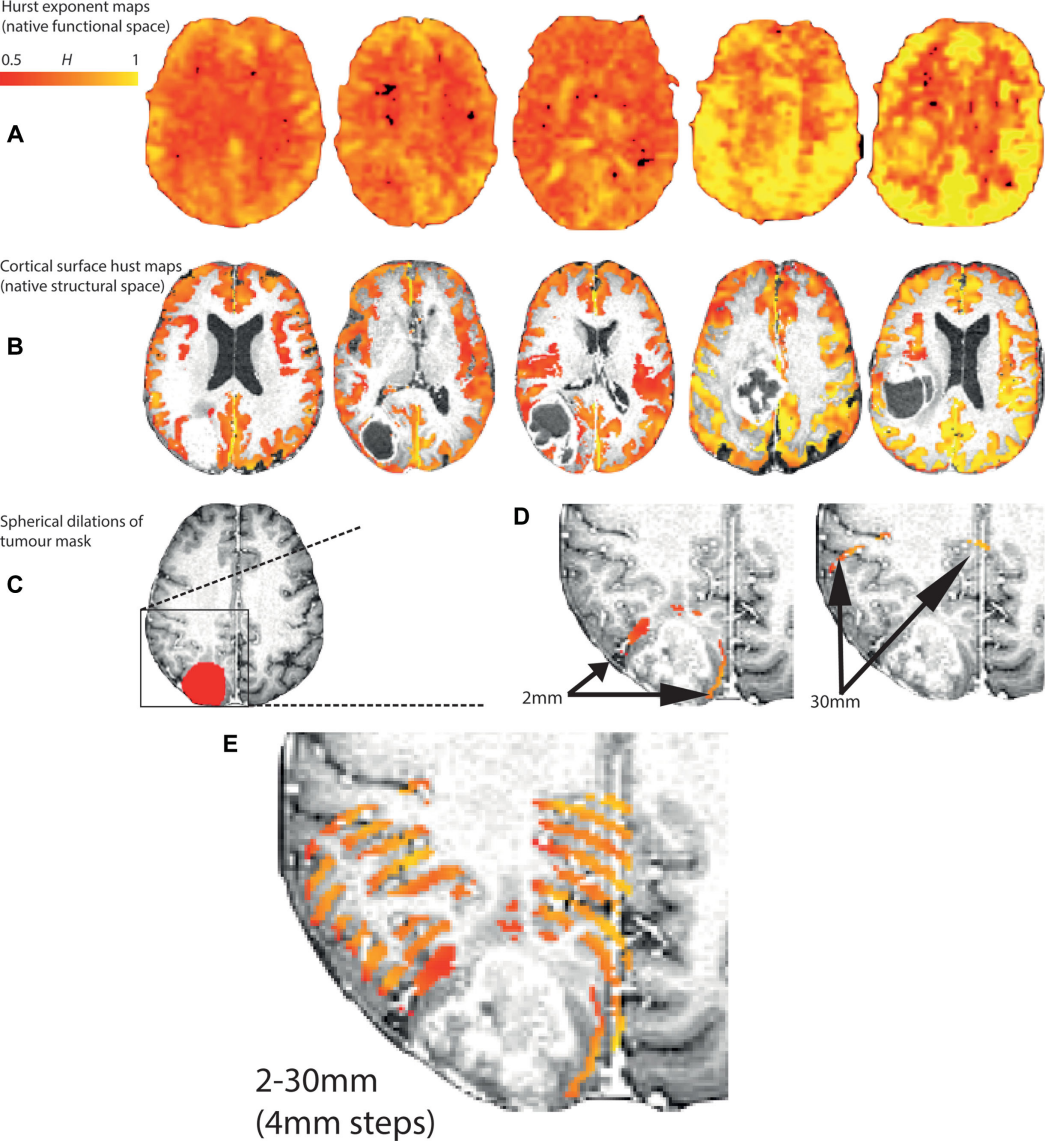
Sequential mask dilations. **A**, Hurst exponent maps (3D volumes) for each patient in the discovery cohort. **B**, Brain extracted and intensity normalized structural images corresponding to each individual. Overlaid are the cortical thickness maps (generated in ANTS) that are used to mask the raw Hurst exponent maps (in structural space) ie, the Hurst exponent is extracted specifically for the grey matter. **C**, A detail from a single patient with an example binary tumor mask drawn in structural space. **D**, The tumor mask subsequently underwent sequential 2 mm dilations with subtraction of the previous tumor mask to produce a 2-mm thick tumor rim. This dilating tumor rim is used to extract H values within the cortex. **E**, The image on the right shows the results of dilations at 2, 6, 10, 14, 18, 22, 26, and 30 mm.

The hand drawn binary tumor masks were dilated to 30 mm beyond their original margins in 2-mm increments, followed by the subtraction of the preceding masks. This created a series of 2-mm annuli expanding from the tumor margin. These annuli were then masked, only being included in further analyses where they overlapped the cortical grey matter estimated from the structural MRI (Figure 3). For each mask, for each participant, average H estimated from fMRI was extracted for each annular mask. Masks were subsequently reflected across the interhemispheric fissure to compute the corresponding values from the contralateral, healthy hemisphere. Values were then expressed as the ratio of H in homologous regions.

### Connectomics

A summary of the connectome analysis pipeline is presented in Figure 1. Parcellation was initially performed with an anatomical template of 251 equal sized parcels^31^ and then subsequently all analyses were repeated on a randomized template of 256 equal sized parcels^32^ to test for parcellation independence of network features. Parcels overlapping the tumor mask were removed from subsequent processing. Statistical dependencies between mean processed time series of each parcel were computed using wavelet, Pearson, and partial correlations to test for independence of network features from the underlying method of connectome constructions. Negative correlations were excluded but thresholding was not applied (ie, matrices were ‘fully connected’).

A graph is composed of nodes (brain regions) and links, which are associated with weights that denote the strength of the connection between nodes. Specific graph theoretical measures were specified pre-hoc, chosen to reflect fundamental centrality features of the underlying network, including node strength; global efficiency; betweenness centrality^33^; within-module degree z-score^34^; participation coefficient^34^; eigenvector centrality.

A network measure particularly pertinent to neurosurgery is node robustness. This is tested by synthetic lesioning, whereby a node (and all of its connections) is removed from the network and the percentage change (delta) of a selected global network measure is computed. Typically, this is performed with global efficiency in which case the resultant measure is also known as delta efficiency. Each node's delta efficiency values were expressed as a ratio over the value in the homologous node in the contralateral hemisphere. Euclidean distance was defined from the center of each node to the center of the tumor. Network measures were computed using the brain connectivity toolbox and transformed to Z-scores.^35^

### Statistics

Between-tissue differences in H were tested with Analysis of Variance (ANOVA), with the threshold for significance, *P* < .05. Testing relationships with distance for the dilating tumor mask and H parcellation analyses were performed with linear ( *Pk* = a*ek*b), and exponential including intersection ( *P* (*k*) = *a* + b*e^k*c^) models. Goodness of fit was determined with the Akaike Information Criteria.^36^ Given the distinct recruitment criteria used for the discovery and validation cohorts, differences in the association between log-transformed H and tumor distance where tested using an Analysis of Covariance (ANCOVA) test. All correlations between network measures underwent Bonferroni correction for multiple comparisons.

## RESULTS

### Tissue Hurst Values

**FIGURE 4.**
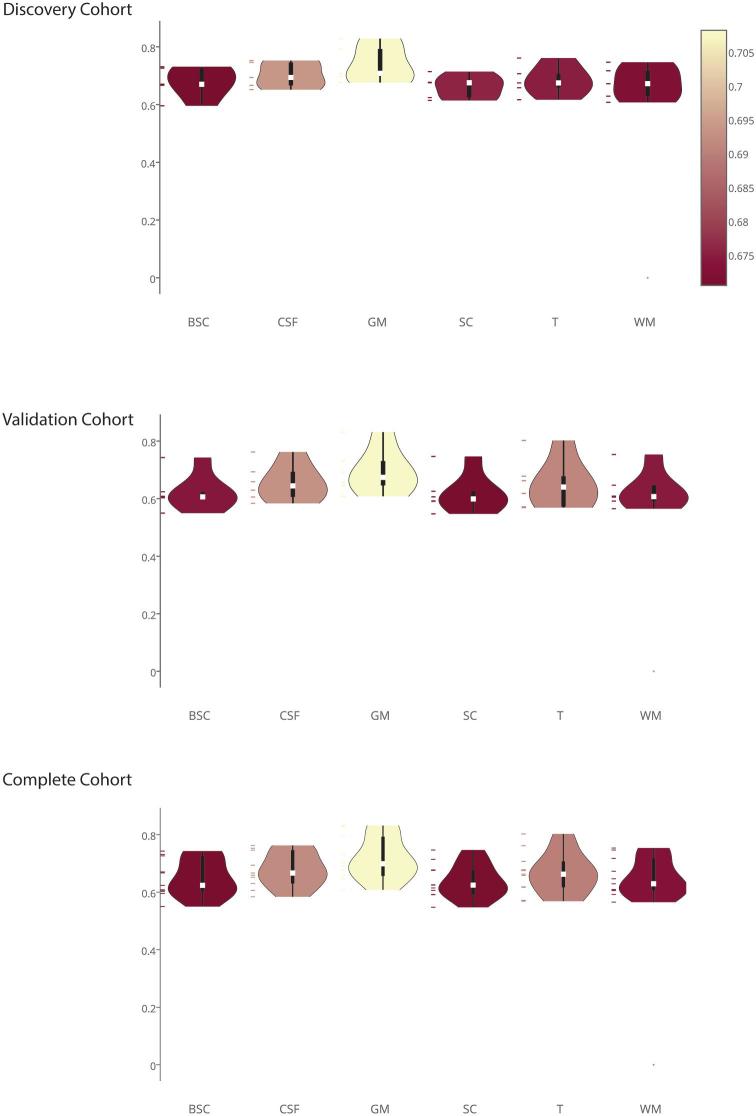
Hurst tissue segmentations. Box plots of extracted H group mean values for tissue segmentations of discovery, validation, and complete cohorts including grey matter, white matter, subcortical structures, contrast enhancing tumor volume, and infratentorial structures (brain stem and cerebellum). Median H is denoted by the white square and color-coded, boxes represent interquartile ranges, and whiskers represent 1.5 times the interquartile range. BSC = brainstem and cerebellum, CSF = cerebrospinal fluid, GM = grey matter, SC = subcortical nuclei, T = tumor, WM = white matter.

Whole-brain Hurst exponent maps, and H masked by cortical segmentation estimated with ANTs from the structural MRI of the discovery cohort, are presented in Figure 3. Brain extraction, registration and segmentation with ANTs resulted in high-quality images using default parameters without noticeable artifacts due to the presence of the tumor. Differences in the means of the Hurst exponent between neocortical grey matter and white matter for the discovery, validation, and complete cohorts were not significant (ANOVA, F = 1.28, *P* = 0.31, Figure 4), although the observed higher values in grey, relative to white matter are consistent with the previous findings.^18^

### Peritumoral Hurst Effects With Distance

**FIGURE 5.**
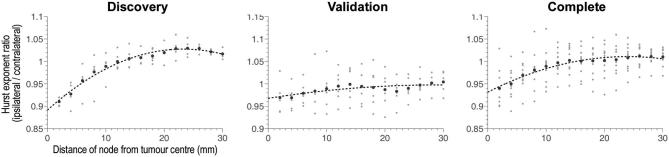
Hurst exponent versus distance from tumor center in dilating tumor masks. The mean H value is plotted as a function of distance from the tumor border in 2 mm increments up to a maximum of 30 mm. The cohort is split into discovery, validation, and complete cohorts from left to right, all of which demonstrate significant quadratic fits (Table 2).

Using the tumor mask sequential dilation technique revealed a quadratic increase in H with distance from the tumor (R^2^ = 0.98, *P* < 10^−4^, AIC = −18.2, Figure 5 left) in the discovery cohort. The quadratic increase in H was replicated in both, the validation (R^2^ = 0.65, *P* < 10^−3^, AIC = −17.2; Figure 5 middle) and complete (R^2^ = 0.95, *P* < 10^−4^, AIC = −18.3; Figure 5 right) cohorts. The ANCOVA analysis revealed an interaction effect between log-transformed H and tumor distance (F = 11.7, *P* < 0.0001) across cohorts. This association was significantly weaker for the validation cohort than for the discovery and complete cohort (*P* < 0.05; post-hoc Bonferroni corrected). A penumbra of reduced complexity relative to the contralateral hemisphere extended for 10 to 15 mm from the tumor border, while beyond 15 mm complexity continues to increase beyond the values seen contralaterally, albeit nonmonotonically, suggesting a complex relationship to global changes.

### Whole Brain Parcellation of Hurst Versus Distance

**FIGURE 6.**
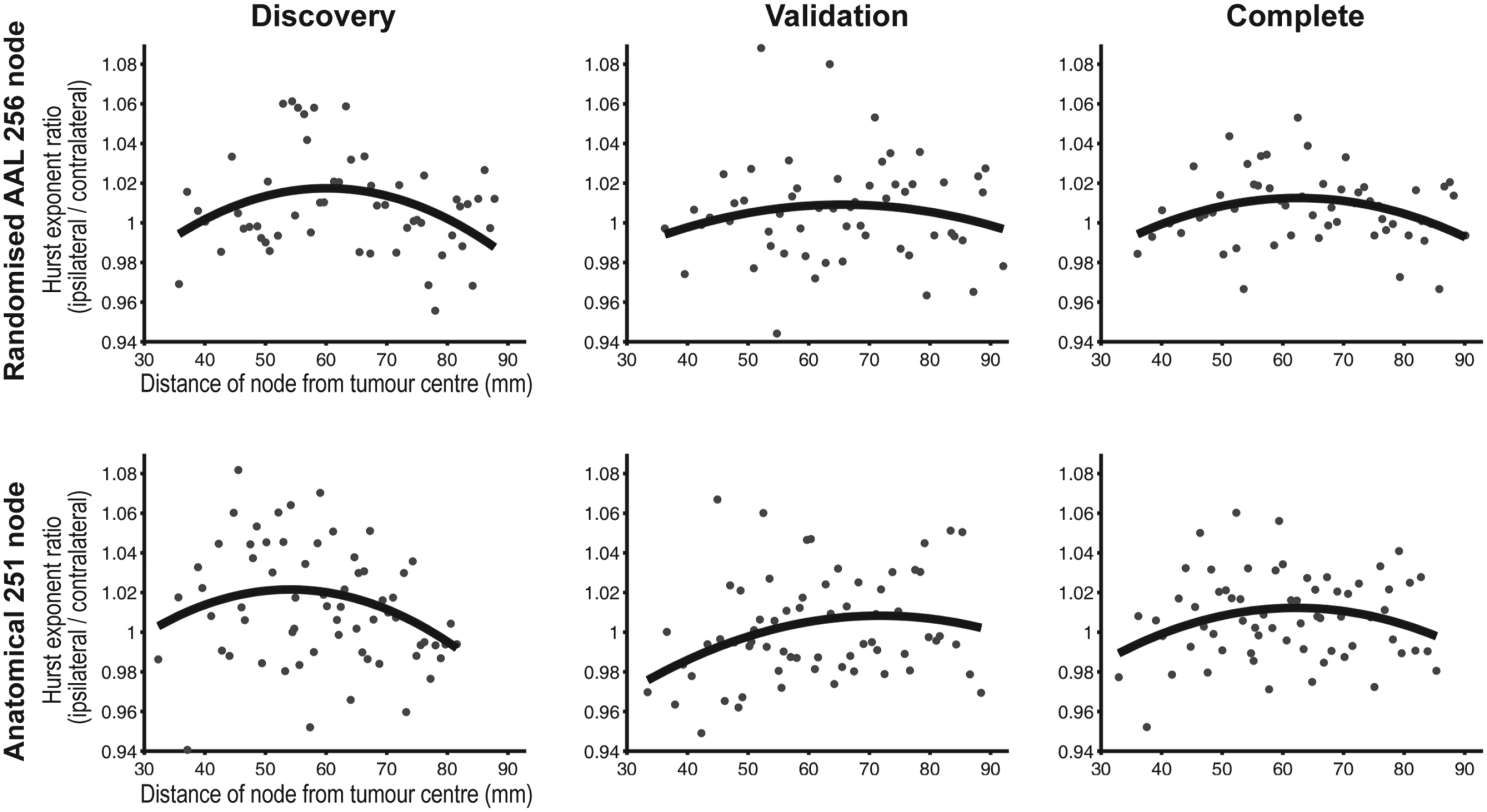
Hurst exponent versus distance in parcellation nodes. The ratio of H between homologous parcels in each hemisphere is plotted against distance from the tumor center. Both parcellation templates are represented (top row = randomized, lower row = anatomical). Participants are split into discovery, validation, and complete cohorts from left to right. The quadratic model demonstrated the best fit for all plots and is highlighted.

To expand on the changes in H related to the distance from the tumor border, the H maps were parcellated using the same templates as for the connectome analysis, with the value of each parcel reflecting the mean H. This allowed greater coverage and a whole brain analysis to complement the limited range of the dilating spherical mask, but with coarser resolution than the dilating tumor mask approach. Results were consistent with those from the tumor mask sequential dilation technique in that closest to the tumor values were reduced compared with the contralateral hemisphere, and then increased with distance to above contralateral levels, before finally reducing back to contralateral values (Figure 6). This trend was best fit with a quadratic model (R^2^ 0.07–0.12, AIC −9.77 to −12.2; Table 2) in the discovery cohort. The validation cohort also demonstrated this quadratic variation in H with distance from the tumor, again demonstrating replicability as with the sequential mask dilation technique. Furthermore, results were independent of the parcellation technique, with both anatomical and randomized parcellation templates demonstrating the same quadratic variation in H with distance from the tumor in all three cohorts (discovery, validation, and complete). These apparently modest R values reflect that distance is but one of likely many factors driving variation in H. Additionally, the magnitude of these changes is broadly comparable with percentage changes in task-based functional MRI, where signal changes of 2%−4% for primary cortices and 1%−2% for association cortices are typically quoted.

**TABLE 2.** Hurst Versus Distance Model Comparisons.

	Anatomical parcellation						Randomised parcellation					
	Discovery		Validation		Complete		Discovery		Validation		Complete	
Fitting	R^2^	AIC	R^2^	AIC	R^2^	AIC	R^2^	AIC	R^2^	AIC	R^2^	AIC
Linear	0.02	−9.70	0.056	−10.48	0.004	−11.15	0.02	−10.80	0.00	−10.55	0.005	−12.04
Quadratic	**0.07**	−**9.77**	**0.085**	−**10.51**	**0.057**	−**11.23**	**0.12**	−**10.99**	**0.02**	−**10.56**	**0.099**	−**12.20**
Cubic	0.06	−9.72	0.084	−10.48	0.052	−11.19	0.11	−10.92	0.02	−10.52	0.091	−12.15
Exponential	0.02	−9.70	0.055	−10.48	0.004	−11.15	0.02	−10.80	0.00	−10.55	0.005	−12.04
Exponential + Intersect	0.02	−9.67	0.055	−10.45	0.003	−11.12	0.02	−10.76	0.00	−10.51	0.001	−12.00

The best fitting model in terms of maximal AIC is highlighted in bold typeface. R^2^ = coefficient of determination, AIC = Akaike Information Criteria.

### Whole Brain Network Measures Distance Effects

**FIGURE 7.**
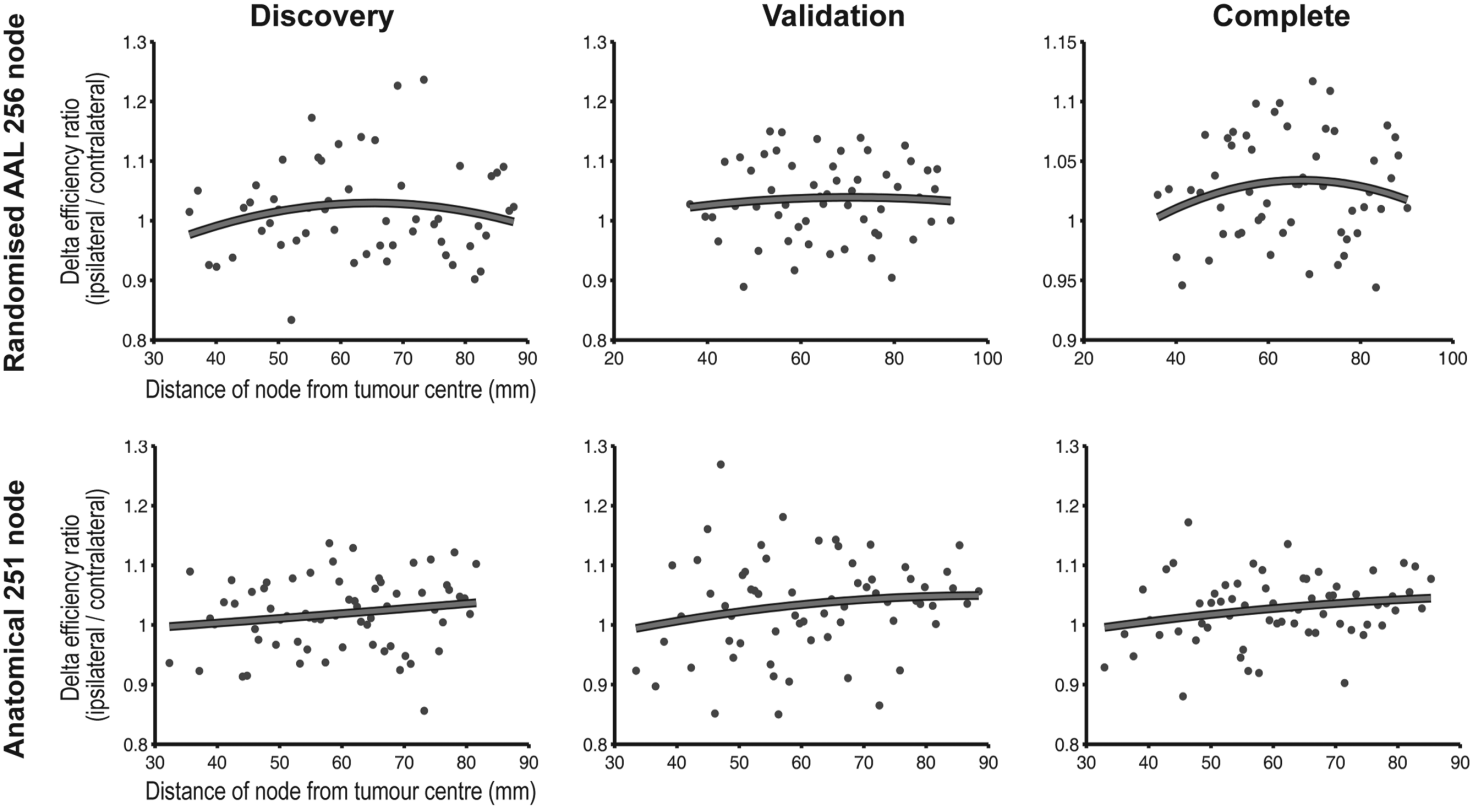
Network efficiency versus distance. Top row images represent randomized parcellation template, lower row represents anatomical parcellation template, both for networks constructed with Pearson correlations. No consistent significant fits were demonstrated. Similar results are present with wavelet and partial correlation networks.

To further expand upon the distance-related changes in H, we investigated whether functional connectome graph theory measures also demonstrated complimentary distance-related effects, specifically that of network robustness. No significant distance-related trends, linear or quadratic, were identified for any graph theory measure (Figure 7). Again, results were consistent across discovery and validation cohorts, anatomical and randomized parcellation templates, and with links weighted by either Wavelet or Pearson product-moment correlations.

### Network Measures Relationship to Hurst Exponent

**FIGURE 8.**
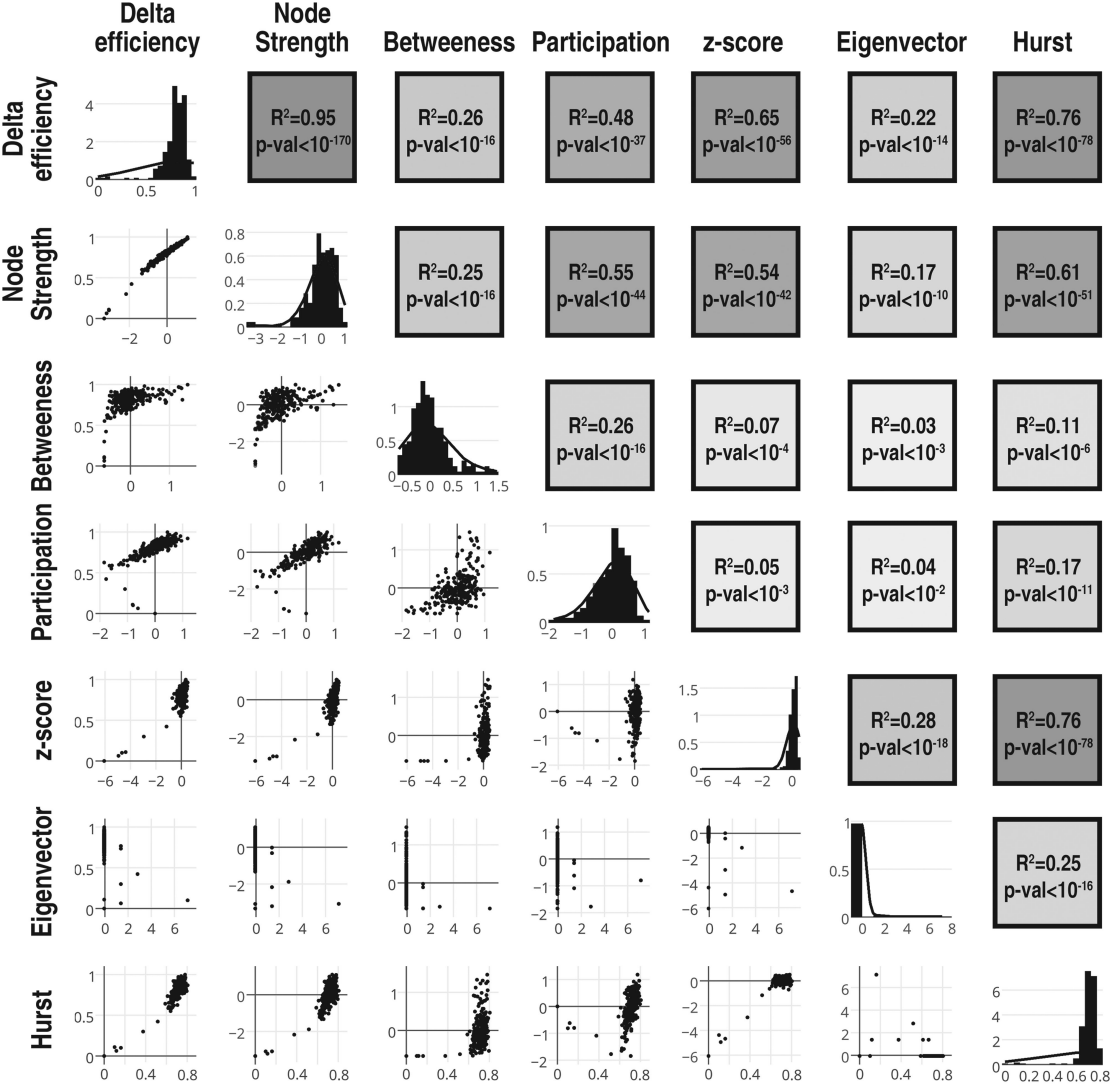
Network measures compared to Hurst exponents. Plotmatrix of selected network centrality measures and H. Diagonal elements represent the histograms of each value after z-transforms. Above diagonal elements represent the corresponding R^2^ and P values as a heatmap.

Finally, given that node H, but not graph theory measures were related to distance from the tumor margin, the relationship between H of resting state BOLD time series and functional connectome graph theory measures was explored. H was significantly correlated (r = 0.33-0.87, *P* < 10^−6^) with all selected network measures, although distributions were negatively skewed (Figure 8).

## DISCUSSION

In this paper, combined fractal and connectomic analyses were performed in a cohort of participants with brain tumors to determine whether the functional sequelae of focal lesions are apparent at the global rather than just the local level. Fractal scaling showed long-range gradients with distance from the tumor margin that are best fit by a quadratic model that describes proximal suppression of complexity, followed by a subsequent increase, before final normalization to values similar to those in the contralateral hemisphere. Furthermore, there was a correspondence between fractal properties of the BOLD signal and network measures, specifically those corresponding to centrality and network robustness. Together these findings give credence to the novel hypothesis that the brain responds to focal tumors by reconfiguring its complexity globally, or at least far beyond the tumor margin. Previously, functional localization has been based on the premise that focal injury is responsible for the resultant phenotype; this work suggests that the functional sequelae of focal brain tumors, and the resultant clinical effects, need to be interpreted in terms of spatially extended changes to whole-brain functional complexity and network architecture.

In the context of BOLD signal resting state functional MRI, a higher value of H represents increased complexity of the time series and better neuronal function (ie, a more complex signal is able to carry more information and hence represents increased processing capacity). This has been corroborated in published studies commenting on the relationship between H and cognitive function, neurodegeneration, and pharmaceutical enhancement of performance.^17-19,37^ In this particular instance, the reduction in the Hurst exponent in the peritumoral region could represent a penumbra of regional cortical suppression, which may arise from direct tumor invasion or distant electrochemical effects.

Correlation between centrality measures and network robustness implies the peritumoral suppression of H relates to brain tissue that has already had role reduction within the network. In terms of extended surgical resections, this suggests that removal of the surrounding peritumoral brain might be tolerated at the functional and network levels, and might not result in significant additional functional impairment. Conversely, if the tumor was mediating an electrochemical suppressant affect, focused removal of only the lesion may ‘release’ this suppression and facilitate functional recovery. Key questions are regarding the clinical effects of this phenomena are how it relates to function and impairment, and whether it recovers after removal of the contrast enhancing tumor mass.

In this analysis, no distance-related effects were found for any graph theory measure, but they were in turn significantly correlated with H. This discrepancy in distance-related effects for H and network measures could of course be related to a true lack of correspondence between them, or because the distance-related effects themselves only partially explain the overall variance in the data. H is a local measure of BOLD signal complexity, and this appears to be altered in a way that is consistent across patients. However, these nonlinear distant-related changes to brain physiology may impact graph theoretical measures of centrality that characterize topological properties over spatially extended brain circuits in a more individual manner, diluting consistent distance-related effects.

Previous applications of resting state fMRI to neuro-oncology have concentrated on mapping primary cortices and determining correspondence with awake brain stimulation findings. Here we develop these methods in performing whole brain mapping that can find general rules about the brain's reaction to the presence of focal lesions. Task-based functional MRI has developed an established role in clinical practice yet often notes activations beyond that which one would expect a priori.^10^ Our data suggest that tumors have divergent effects on the brain depending on distance, implying that multiple mechanisms may be represented by such unexpected activations and not just plasticity.

Effects of focal cerebral lesions on cognition are determined by a number of factors,^38,39^ while detailed cognitive examination often reveals highly variable findings between patients despite broadly similar histological and demographic characteristics.^40^ Understanding the peritumoral brain at the individual level will be critical to determining the safety of resections, or indeed if it is possible to safely exceed the margins of a lesion in an effort to increase survival. Fractal analysis could be used to identify regions of suppressed cortical function that could be safely resected, or indeed areas that may improve upon resection of the main tumor volume and should be left untouched.

This study is a proof-of-concept investigation into the effects of focal tumors on the complexity of BOLD signals and the functional connectome at the global level. While the overall number of participants was limited, it was felt the advantages of dividing the cohort into discovery and validation groups, namely increased methodological robustness and the ability to test replicability of findings, outweighed any marginal increase in statistical power from analyzing participants in a single cohort. This resulted in relatively small cohorts, but with the benefit of each participant acting as their own control this was not deemed to preclude analysis, particularly given the goal was to identify robust findings at the global level.

High-grade gliomas have previously been noted to have complex effects on the hemodynamic response function and BOLD contrast.^10^ We sought to mitigate any potential artifacts with a robust preprocessing strategy including ICA-based denoising. Furthermore, the robust distance-related effects described here suggest an alternative hypothesis whereby the previously described peritumoral effects on BOLD contrast are at least partially related to local cortical suppression rather than hemodynamic or oxygenation abnormalities. Validation of these findings in a cohort of participants with low grade glioma, where the effects on BOLD contrast are not apparent, would further increase the reliability of these findings and is currently being undertaken in a follow-up study. Multimodal imaging (for example, also including FLAIR, Multiparametric Mapping, and NODDI protocols) will be invaluable to fully defining the individual characteristics of specific tissues.

A rational follow-up to this work would be to include cognitive and longitudinal follow-up data to test the generated hypotheses; namely, that cognitive sequelae are dependent on the distance of resection from the tumor margin. Cognitive and longitudinal outcome data will aid inference on the compensatory or decompensatory nature of long-range functional gradients. Finally, larger heterogeneous cohorts will help determine whether these functional effects are consistent or if there are specific drivers underlying individual variation in gradients of changes.

## CONCLUSION

Fractal and connectomic analyses suggest global rather than local effects of focal tumors on brain function. Furthermore, exploration of fractals and complex network measures revealed a strong relationship between signal complexity and graph theory measures, notably those reflecting network robustness. It is therefore conceivable that extent of resection, a key prognostic marker for survival, could be approached as an optimization problem balancing increased tumor removal with fractal scaling and network robustness. Further data could be used to test hypotheses that these distance-related effects reflect brain plasticity and potentially allow extended resection of the tumor without functional cost.

### Disclosures

Dr Hart is funded by the Wellcome Trust Neuroscience in Psychiatry Network with additional support from the National Institute for Health Research Cambridge Biomedical Research Centre. The imaging studies were funded by an NIHR Clinician Scientist Fellowship for Dr Price (NIHR/CS/009/011). The views expressed are those of the authors and not necessarily those of the NHS, the NIHR, or the Department of Health. The authors have no personal, financial, or institutional interest in any of the drugs, materials, or devices described in this article.

## COMMENT

This study seeks to address an important question in neurosurgery: what is the brain-wide effect of a focal lesion? From clinical experience, we know that mass lesions can have an effect on neurological function that seem broader than would have been predicted by the size and location of the lesion. In this paper, the authors describe their attempts to apply quantitative techniques to this question in 11 patients with GBM. Their fractal analysis of resting state fMRI data suggest an inverted U-shaped relationship between distance from the tumor and a measure of neural complexity/functionality. The implication proposed by the authors is that peritumoral tissue, which has lower than typical complexity/functionality, already has a reduced role in the network. If so, then removal of this peritumoral rim may not produce significant further functional impairment. Other interpretations of the results, however, are certainly possible, and could lead to different management strategies. It would seem likely that this measure will depend upon where the lesion is (eg, near or far from known functional anatomical boundaries), and how it affects blood flow, which is the basis for resting state fMRI signals (eg, AVM vs high-grade vs low-grade tumor).

As with any new analysis method applied to clinical questions, further experience and data will be necessary to understand the meaning of these results. The dependent variable, an estimate of the Hurst exponent, is a complex mathematical construct involving a model based on the logarithm of variances of coefficients of wavelet decompositions of resting state fMRI data. Needless to say, this quantity is heavily abstracted from clinical variables, but is taken to represent some measure of brain function. It will take much more work to understand whether this measure has predictive or explanatory power with biological significance. I congratulate the authors for their initial foray in this complex space. This preliminary work has set the stage and will hopefully encourage others to apply sophisticated computational analyses to questions of great neurosurgical relevance.

**Sameer A. Sheth**

*Houston, Texas*
